# Lung UltrasouNd Guided surfactant therapy in preterm infants: an international multicenter randomized control trial (LUNG study)

**DOI:** 10.1186/s13063-023-07745-8

**Published:** 2023-11-04

**Authors:** Iuri Corsini, Javier Rodriguez-Fanjul, Francesco Raimondi, Luca Boni, Alberto Berardi, Victoria Aldecoa-Bilbao, Almudena Alonso-Ojembarrena, Gina Ancora, Salvatore Aversa, Renzo Beghini, Nerea Bilbao Meseguer, Letizia Capasso, Francesca Chesi, Martina Ciarcià, Ana Concheiro, Luigi Corvaglia, Benjamim Ficial, Luca Filippi, Jesus Fuentes Carballal, Monica Fusco, Sara Gatto, Gemma Ginovart, Rebeca Gregorio-Hernández, Gianluca Lista, Manuel Sánchez-Luna, Silvia Martini, Luca Massenzi, Francesca Miselli, Domenica Mercadante, Fabio Mosca, Marta Teresa Palacio, Alessandro Perri, Francesca Piano, Marcelino Pumarada Prieto, Lorena Rodeno Fernandez, Francesco Maria Risso, Marilena Savoia, Alex Staffler, Giovanni Vento, Carlo Dani

**Affiliations:** 1 Division of Neonatology, Careggi University Hospital of Florence, Largo Brambilla 3, 50134 Florence, Italy; 2 Neonatal Intensive Care Unit. Hospital Germans Trias I Pujol, Badalona, Spain; 3 Division of Neonatology, Federico II University of Naples, Naples, Italy; 4 SC Epidemiologia Clinica IRCCS Ospedale Policlinico San Martino, Genoa, Italy; 5 Neonatal Intensive Care Unit, Policlinico Universitario Modena, Modena, Italy; 6 Neonatal Intensive Care Unit, Hospital Clínic Barcelona, BCNatal (Barcelona Center for Maternal Fetal and Neonatal Medicine), Barcelona, Spain; 7 Neonatal Intensive Care Unit, Hospital Puerta del Mar, Cadiz, Spain; 8 Neonatal Intensive Care Unit, Ospedale Infermi Di Rimini, Rimini, Italy; 9 Neonatal Intensive Care Unit, Children’s Hospital, ASST Spedali Civili, Brescia, Italy; 10 Neonatal Intensive Care Unit, AOUI Verona, Verona, Italy; 11 Neonatal Intensive Care Unit, Hospital Basurto, Bilbao, Spain; 12 Neonatal Intensive Care Unit, Department of Clinical and Experimental Medicine, University of Pisa, Pisa, Italy; 13 Neonatal Intensive Care Unit, Hospital Alvaro Cunqueiro, Vigo, Spain; 14 Neonatal Intensive Care Unit IRCCS AUOBO, Department of Medical and Surgical Sciences, University of Bologna, Bologna, Italy; 15 Neonatal Intensive Care Unit, Complexo Hospitalario Universitario de A Coruña (CHUAC), Coruña, Spain; 16 Neonatal Intensive Care Unit, Ospedale Dei Bambini “V.Buzzi”, Milan, Italy; 17 Neonatal Intensive Care Unit, Hospital Gregorio Marañon, Madrid, Spain; 18 Neonatal Intensive Care Unit, Bolzano, Italy; 19 Neonatal Intensive Care Unit, Fondazione IRCCS Ca’ Granda Ospedale Maggiore Policlinico, Milan, Italy; 20 Department of Clinical Sciences and Community Health, University of Milan, Milan, Italy; 21 Neonatal Intensive Care Unit, Policlinico Gemelli, Rome, Italy; 22 Neonatal Intensive Care Unit, Udine, Italy

**Keywords:** Preterm infants, Lung ultrasound, Respiratory distress syndrome, Surfactant therapy

## Abstract

**Background:**

The management of respiratory distress syndrome (RDS) in premature newborns is based on different types of non-invasive respiratory support and on surfactant replacement therapy (SRT) to avoid mechanical ventilation as it may eventually result in lung damage. European guidelines currently recommend SRT only when the fraction of inspired oxygen (FiO_2_) exceeds 0.30. The literature describes that early SRT decreases the risk of bronchopulmonary dysplasia (BPD) and mortality. Lung ultrasound score (LUS) in preterm infants affected by RDS has proven to be able to predict the need for SRT and different single-center studies have shown that LUS may increase the proportion of infants that received early SRT.

Therefore, the aim of this study is to determine if the use of LUS as a decision tool for SRT in preterm infants affected by RDS allows for the reduction of the incidence of BPD or death in the study group.

**Methods/design:**

In this study, 668 spontaneously-breathing preterm infants, born at 25^+0^ to 29^+6^ weeks’ gestation, in nasal continuous positive airway pressure (nCPAP) will be randomized to receive SRT only when the FiO2 cut-off exceeds 0.3 (control group) or if the LUS score is higher than 8 or the FiO2 requirements exceed 0.3 (study group) (334 infants per arm). The primary outcome will be the difference in proportion of infants with BPD or death in the study group managed compared to the control group.

**Discussion:**

Based on previous published studies, it seems that LUS may decrease the time to administer surfactant therapy. It is known that early surfactant administration decreases BPD and mortality. Therefore, there is rationale for hypothesizing a reduction in BPD or death in the group of patients in which the decision to administer exogenous surfactant is based on lung ultrasound scores.

**Trial registration:**

ClinicalTrials.gov identifier NCT05198375. Registered on 20 January 2022.

## Background

The management goal of neonatal respiratory distress syndrome (RDS) is to improve survival of affected neonates using non-invasive respiratory support, surfactant therapy, mechanical ventilation, and overall care of the premature neonate [1].

Currently, the European consensus guidelines on RDS recommend administering surfactant in non-invasively ventilated neonates when the fraction of inspired oxygen (FiO_2_) is higher than 0.30 during nasal continuous positive airway pressure (nCPAP) of at least 6 cmH_2_O [1]. Since oxygenation depends on both FiO_2_ and mean airway pressure, the accuracy of this criterion might be suboptimal because the setting of respiratory support is not clearly standardized. Moreover, there is a large body of evidence that early exogenous surfactant administration plays a pivotal role in the treatment of RDS. In fact, in the short term, it reduces the incidence of pneumothorax. In the long term, it improves survival and, since it may allow the avoidance of invasive mechanical ventilation, minimizes the development of bronchopulmonary dysplasia (BPD) and death [2].

On the contrary, waiting to fulfill FiO_2_ criteria for surfactant replacement therapy (SRT) can lead to delayed administration and reduce the potential beneficial effects of surfactant [3, 4]. At present, there is no universal consensus on the criterion and cut-off to adopt for surfactant treatment [1, 5, 6].

The role of lung ultrasound (LU) as a semi-quantitative method to decide whether to administer exogenous surfactant has been extensively studied in recent years. The first study by Brat et al. [7] demonstrated that a LU score (LUS) quantifies RDS severity and can predict the need for SRT. This was subsequently confirmed by De Martino et al. [8]. Following these preliminary results, Raschetti et al. [3] demonstrated in a single-center quality-improvement study that the use of a LUS cut-off to guide SRT led to a significant increase of the proportion of neonates who received surfactant treatment in the optimal therapeutic window (defined as therapy carried out within the first 180 min of life), without increasing the number of treated infants. This finding has been confirmed by a recent single-center randomized controlled trial by Rodriguez-Fanjul et al. [4]. Although the adoption of LUS can enhance the quantitative diagnostic evaluation of severity of RDS and allows early identification of neonates who will later require SRT, there is not enough evidence that this significantly leads to a clinical benefit; the universal use of LUS to guide surfactant treatment outside a research setting still cannot be supported. To the best of our knowledge, there are no multicenter randomized controlled trials which evaluate short- and long-term clinical outcomes in preterm infants who receive echo-guided surfactant treatment in comparison with current treatment based on the FiO_2_ criterion.

### Trial hypothesis

We hypothesized that the use of a LUS to quantify RDS severity and guide surfactant treatment (study group) may increase the proportion of preterm infants receiving timely SRT and eventually improve their short- and long-term outcomes in comparison with a control group treated according to the FiO_2_ criterion alone.

Therefore, our hypothesis is that the study group will show a decrease in the proportion of infants affected by BPD or death.

To confirm this hypothesis, we planned an international multi-center randomized controlled study in which preterm infants are randomized into two groups: one will receive SRT based on the FiO_2_ criterion and the other will receive SRT on the basis of LUS and/or FiO_2_ cut-off.

**Fig. 1:**
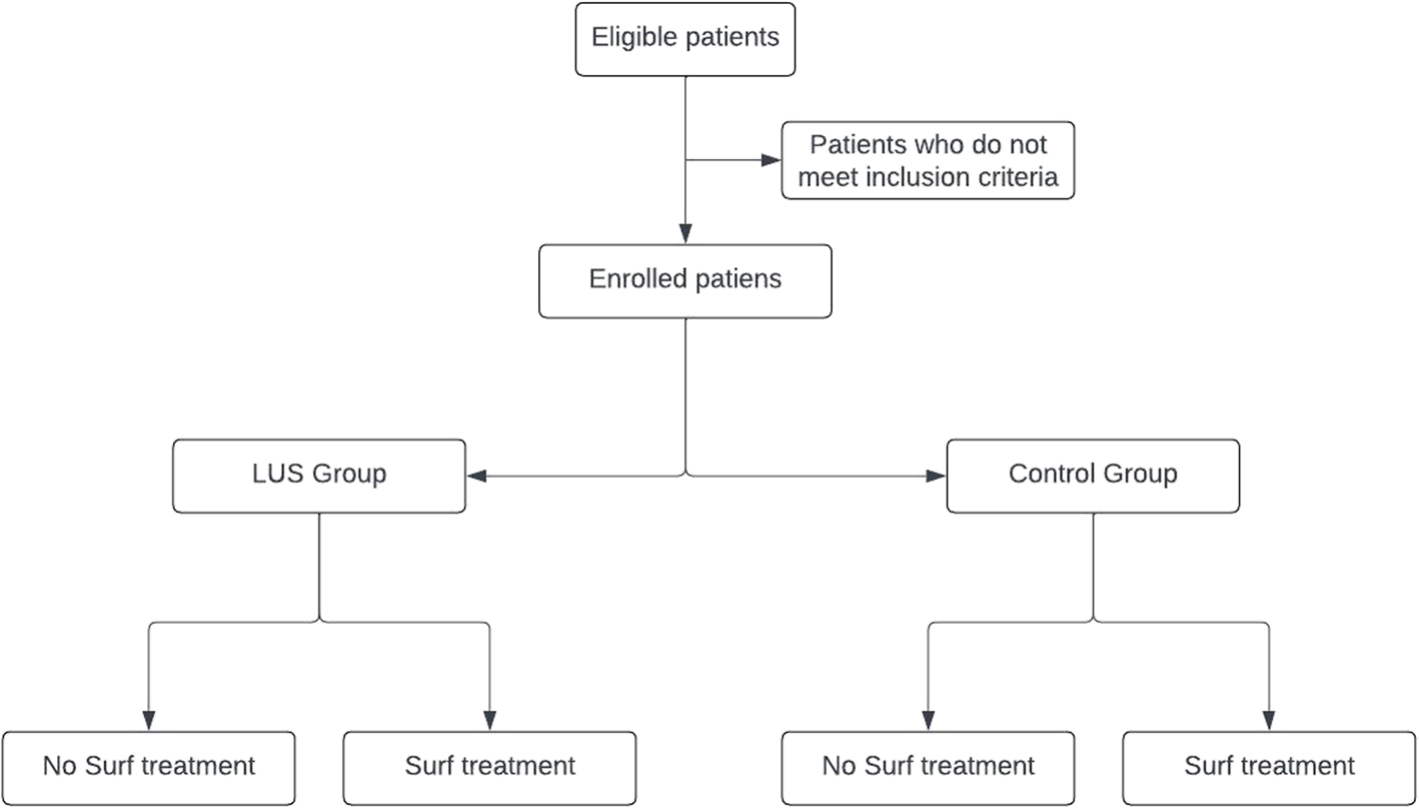
Study flow chart

The study flow chart is detailed in Fig. 1.

### Roles and responsibilities for committees

LUNG study is led by a Steering Committee comprising the senior investigator (CD), the national coordinators (IC for Italy and JRF for Spain), and two national experts (FR for Italy and MSL for Spain) who will oversee the progress and adherence of the centers to the study protocol. There will be one principal investigator in each center who will be responsible for obtaining ethical approval, organizing local Good Clinical Practice monitoring and data entry into the patient report forms.

## Methods/design

### Study design

This will be an unblinded multi-center randomized open control trial with two parallel groups of surfactant treatment based on FiO_2_ cut-off versus LUS and/or FiO_2_ cut-off in spontaneously breathing infants in nCPAP for RDS born at 25^+0^ to 29^+6^ weeks of gestational age (GA).

The financial outcome of the study is no profit. The design of the study is of superiority.

### Participating centers

The following centers are actively recruiting for the trial:

- Careggi University Hospital of Florence, Florence, Italy; Germans Trias i Pujol, Badalona, Spain; Federico II University of Naples, Naples, Italy; Udine Hospital, Italy; Fondazione IRCCS Ca' Granda, Milano, Italy; Brescia Hospital, Italy; Pisa University, Italy; IRCCS AUOBO University of Bologna, Bologna, Italy; Policlínico Gemelli, Rome, Italy; Gregorio Marañon, Madrid, Spain; Basurto, Bilbao, Spain; Puerta del Mar, Cadiz, Spain; Alvaro Cunqueiro, Vigo, Spain; Clinic, Barcelona, Spain; Complexo Hospitalario Universitario de A Coruña (CHUAC), Spain; Ospedale Infermi di Rimini, Rimini, Italy; Policlinico Universitario di Modena; Bolzano Hospital, Ospedale dei Bambini “V. Buzzi” Milan, Italy.

We estimate that the participating centers will have a minimum of 10 and a maximum of 70 eligible patients during the study period in order to have a final sample size 668 children (334 per arm). Sample size calculation and trial simulation have been performed with R (see below).

The study will be carried out in third or fourth level NICUs in which medical staff is adequately trained to use LU and quantify RDS severity with LUS.

### Inclusion criteria

Infants fulfilling the following inclusion criteria will be eligible to participate:

1. In-born at 25 + 0 to 29 + 6 weeks of gestational age.
2. Spontaneously breathing at birth but requiring non-invasive respiratory support with nCPAP at a positive pressure of 6–8 cm H_2_O to maintain a peripheral oxygen saturation (SpO_2_) between 90 and 95%.
3. Respiratory distress syndrome (defined as respiratory distress appearing within the first 24 h of life requiring nCPAP to keep peripheral oxygen saturation above 90%, with clinical signs of respiratory difficulty such as polypnea, chest retractions, nasal flutter in the absence of other respiratory diseases non-mandatory criterion was lung images that support the diagnosis [4]).
4. Parental written signed consent has been obtained.

### Exclusion criteria

1. Endotracheal intubation in the delivery room for resuscitation or insufficient respiratory drive according to the European guidelines [1].
2. Prolonged premature rupture of membranes (pPROM) for more than 3 days.
3. Presence of major congenital malformations or chromosomal anomalies.
4. Hydrops fetalis.
5. Inherited disorders of metabolism.
6. Administration of surfactant before performing LU.
7. Air leak syndrome (pneumothorax, pneumomediastinum), congenital diaphragmatic hernia, congenital pneumonia, meconium aspiration syndrome.

### Sample size

The percentage from international databases of infants with GA between 25^+0^ and 29^+6^ weeks expected to die or to develop BPD derived is approximately 40% using the Jobe and Bancalari (2001) definition [9, 10].

In a retrospective analysis of our data, the percentage of composite outcome of BPD and death at participating centers was 35%.

Assuming the percentage of newborns with death or BPD is 35% (the most conservative condition) in the control group and a decrease to 25% in the experimental group, a sample size of 668 children (334 per arm) is required to obtain a statistical power of 80% with alpha 0.05.

An interim analysis will be planned after 167 infants per arm are enrolled (334 total infants enrolled). Based on the results of the interim analysis, the study will continue to be carried out as follows:

- 1: If the statistical *Z* test gives a value equal to or less than 0.01, the study will be terminated for futility;
- 2: If the statistical *Z* test gives a value equal to or greater than 2.75, the study will be terminated for efficacy;
- 3: If the statistical *Z* test gives a value less than 0.41 or higher than 0.8, the study will be continued until the previously calculated sample size is equal to 668.
- 4: If the statistical *Z* test gives a value equal to or higher than 0.41 and equal to or less than 0.8, the final sample size will be modified, but without exceeding the maximum threshold of 1.5 times the expected initial size (sample size max: 1002).

Sample size calculations and trial simulations have been performed with R.

### Randomization

Newborns will be allocated to one of the treatment groups (LUSG or CG) in a 1:1 ratio via a central electronically generated procedure by the e-clintrials platform (https://www.eclintrials.org/ect/) managed by Dr. Luca Boni. The researchers will login to the platform using their personal username and password. Then a randomization form will be completed in order to verify the presence of the inclusion criteria and the absence of the exclusion criteria. Once the patient’s eligibility is confirmed, the portal will carry out an automatic randomization. Randomization is stratified by center and gestational age.

A block randomization method will be used to guarantee an adequate sample number even for the lowest GAs (25^+0^ to 26^+6^ weeks or 27^+0^ to 29^+6^ weeks).

A monthly accrual report about the study will be sent to the participating centers.

### Blinding

This is an open non-blinded study, and the staff performing the study will also take subsequent care of the infants.

### Intervention

Infants will be allocated in one of the two treatment groups in a 1:1 ratio according to the minimization method, using an interactive web-based electronic system.

Infants will be electronically randomized into two groups:

1. Control group (CG): surfactant administration when FiO_2_ > 0.30 on nCPAP (positive pressure 6–8 cmH_2_0) to maintain preductal SpO_2_ between 90 and 95% [1].
2. LUS group (LUSG): surfactant administration when LUS > 8 on nCPAP (positive pressure 6–8 cmH_2_0) to maintain preductal SpO_2_ between 90 and 95% [1].

The LUSG will receive SRT as rescue therapy in case of LUS ≤ 8 but FiO_2_ > 0.30 on nCPAP (positive pressure 6–8 cmH_2_0) to maintain preductal SpO_2_ between 90 and 95%.

The cut-off of LUS > 8 is in agreement with previous studies [3, 4, 11].

### Clinical management

Positive pressure with a neonatal mask and a T-piece system (Neopuff Infant Resuscitator ®, Fisher and Paykel, Auckland, New Zealand) will be used to stabilize the newborns after birth as per routine daily practice. If necessary, infants will start mechanical ventilation in agreement with the European guidelines [1]. In this latter case, subjects will be excluded from the study (see “Exclusion criteria” section).

To better standardize the timing of care after birth, assistance in the delivery room, transfer of the newborn to the NICU, stabilization of the newborn, and the related procedures (positioning of the vascular access, stabilization of ventilatory parameters, thermal homeostasis, etc.) are expected to be completed within the first hour of life.

Once infants with RDS have been screened, enrolled in the study, and assigned to a group (CG or LUSG), LU will be performed as soon as possible between 60 and 180 min of life. In the meantime, patients will be assisted with non-invasive ventilation (nCPAP, positive pressure 6–8 cmH_2_0) and oxygen therapy to maintain preductal SpO_2_ between 90 and 95%. A loading intravenous dose of caffeine citrate (20 mg/kg) will be administered in the first hours of life followed by a maintenance of 5–10 mg/kg/day, as per routine clinical practice.

### Non-invasive ventilation management after the first 180 min of life or after surfactant administration

After the first 180 min of life or once surfactant has been administered, according to the criteria of the randomization group, the newborns can be assisted with nCPAP or other non-invasive ventilation mode (nasal intermittent positive pressure ventilation (NIPPV), bi-level positive airway pressure (BiPAP), high flow nasal cannula (HFNC)) according to the local practice.

### Lung ultrasound procedure

LU will be performed by the attending neonatologist. Centers participating in the study routinely use LU and all the neonatologists are trained for this technique.

**Fig. 2:**
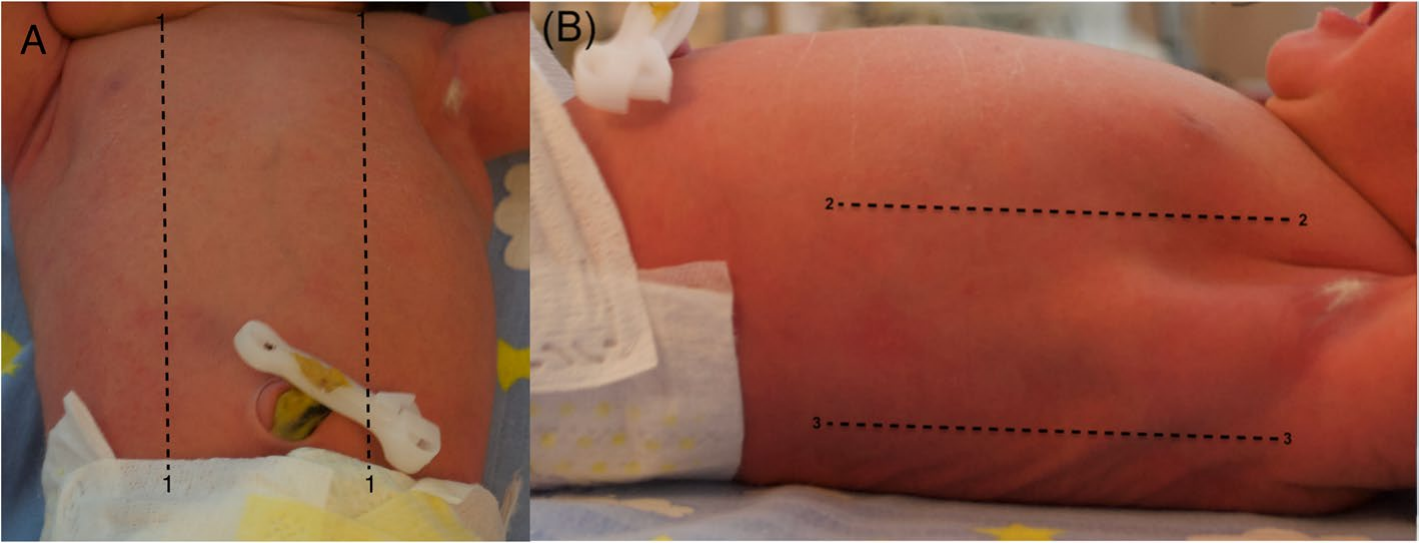
Lung ultrasound score chest partitioning

**Fig. 3:**
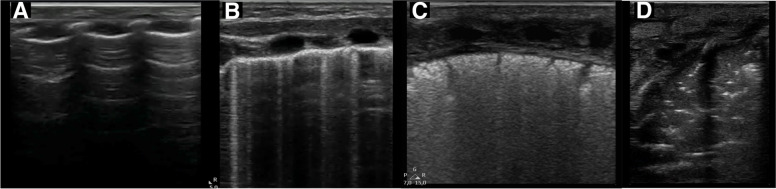
Lung ultrasound area score. Lung ultrasound score for each area is attributed according to the following criteria: 0, A-pattern (defined by the presence of only A-lines; panel **A**); 1, B-pattern (defined as the presence of three or more B-lines, well spaced; panel **B**); 2, severe B-pattern (defined as the presence of crowded and coalescent B lines with or without consolidations limited to subpleural space; panel **C**); and 3, extended consolidation (panel **D**)

LUS will be calculated by performing bilateral longitudinal scans of the chest on the midclavicular, anterior axillary, and posterior axillary line as proposed by Raimondi et al. [12] using high-frequency linear or micro linear (hockey stick) probe (Fig. 2). The focus will be located at the level of the pleural line [13]. A score from 0 to 3 (as proposed by Brat et al. [7]) will be assigned to each scan area based on the ultrasound detected pattern; in case of different score patterns in the same area, the worst will be selected (Fig. 3).

LU can be performed with the patient in supine position without the need of turning as the posterior axillary line is accessible from the side of the newborn [14].

Surfactant replacement therapy should be given if LUS > 8 in subjects recruited in the LUSG.

### Surfactant treatment

Once criteria for surfactant administration have been met, natural surfactant (Poractant alfa, Curosurf ®, Chiesi, Parma, Italy) will be administered (200 mg/kg) according to the InSURE (Intubation-SURfactant-Extubation), LISA (Less-Invasive-Surfactant-Administration), or IN-REC-SURE (INtubation-RECtruitmen-SURfactant-Extubation) technique in both groups as per the enrollment center protocol.

After surfactant administration, patients of both groups will be extubated within 30 min in the case of the InSURE method and IN-REC-SURE method (in presence of satisfactory respiratory drive) and will continue non-invasive ventilation support as per center protocol.

Infants of both groups may receive a subsequent dose of surfactant (100 mg/kg of poractant alfa) using the same method if they fail non-invasive ventilation again during the following 12 to 24 h as per center standard care.

### Analgesia-sedation

Pharmacological premedication with fentanyl and atropine will be allowed (as per center protocol) and recorded.

### nCPAP ventilation failure criteria

In the NICU, nCPAP will be the standard method of non-invasive support in all infants recruited for this trial. nCPAP failure is defined if any of the following criteria are met: FiO_2_ ≥ 0.30 to maintain a SpO_2_ > 90% for at least 30 min unless rapid clinical deterioration has occurred; respiratory acidosis defined as pCO_2_ > 65 mmHg and pH < 7.20 on an arterial or capillary blood gas sample; and apnea defined as more than four episodes of apnea per hour or more than two episodes of apnea per hour which require bag and mask or Neopuff ventilation [15].

### Mechanical ventilation criteria

Mechanical ventilation (MV) should be started if the patient meets one of the following criteria: pCO_2_ > 65 mmHg and pH < 7.20, or paO_2_ < 50 mm Hg, or FiO_2_ > 0.4 after surfactant administration or in case of apnea (> 4 episodes in 1 h or > 2 episodes in 1 h ventilation with bag and mask or Neopuff), and should continue with the aim of maintaining a pCO_2_ of 55–65 mmHg and a SpO_2_ of 90–95%, in conventional MV, or high frequency ventilation (HFV) [15]. Patients will be extubated as per center protocol.

### Concurrent and supportive therapies

The daily treatment practices of patients enrolled in the study will be performed according to the local guidelines of each center. However, to standardize the most relevant procedures in the participating centers, the fluid intake will be based essentially on changes in body weight, serum electrolyte concentration, and serum osmolality starting indicatively with 70–80 mL/kg and increasing by 10–20 mL/kg/day until reaching approximately 150 mL/kg at the end of the first week of life. Maternal or donor milk will be given from the first day of life; if the infusion of a glucose solution is indicated, the concentration of the latter will be chosen in such a way as to maintain appropriate blood sugar levels; electrolytes will be added only after the first day of life, while the intravenous administration of amino acids and lipids will be undertaken from the first day. In the case of systemic hypotension refractory to fluid therapy, vasoactive drugs will be started depending on the underlying pathophysiology.

Newborns will receive antibiotic prophylaxis once blood cultures have been taken, and it will be suspended after 2 or 3 days once results are negative.

Postnatal steroid treatment may be administered in infants with severe respiratory insufficiency under maximal mechanical ventilation and at high risk of mortality or in infants with evolving or established BPD.

### Primary outcome measure

Primary endpoint will be reduction of the proportion of infants with BPD (Jobe and Bancalari (2001) definition) [10] or death in the LUSG versus CG.

### Secondary outcome measures

1. Group comparison of proportion of “early” (before 3 h of life as defined by Raschetti [3] et al. and Rodriguez-Fanjul et al.[4]) vs. late surfactant treatment.
2. Need of MV in the first 3 days of life.
3. FiO_2_ before surfactant treatment.
4. SpO_2_/FiO_2_ ratio before surfactant treatment.
5. Proportion of infants needing surfactant treatment.
6. Duration of non-invasive and invasive respiratory support.
7. Proportion of infants who need multiple doses of surfactant.
8. Pharmacological and/or surgical treatment of patent ductus arteriosus (PDA).
9. Pneumothorax (PNX) rate.
10. Length of stay in hospital.
11. BPD rate (using multiple definition) and severity.
12. Mortality rate.

### Other collected data

For each newborn, the following data will be recorded: gestational age; birth weight; birth weight < 10th percentile; sex; type of delivery; Apgar score at the 5th minute; CRIB score and Silverman score; main pathologies of pregnancy (pre-eclampsia, premature rupture of membranes, clinical chorioamnionitis, placental abruption); mode and parameters of ventilation before SRT; time of surfactant administration; blood gas analysis parameters before SRT; max FiO2 before SRT; minimum SpO2/FiO2 ratio before SRT; type and duration of respiratory assistance (oxygen therapy, nCPAP, BiPAP, HFNC, volumetric ventilation, HFV); antenatal and postnatal steroid treatment; number of surfactant doses; SpO_2_/FiO_2_ ratio at 1, 7 days of life, and at 36 weeks of GA or discharge. In addition, common complications of prematurity will be detected: PDA requiring drug therapy or surgical closure; pneumothorax or other air-leak syndromes; necrotizing enterocolitis (NEC) > 2 grade; BPD; intraventricular hemorrhage (IVH) ≥ 3 grade; periventricular leukomalacia (PVL); retinopathy of prematurity (ROP) of grade > 3; and proven sepsis. Furthermore, mortality and length of stay in NICU will be recorded. BPD diagnosis will be based on the classification of Jobe and Bancalari (2001) [10]. The adapted classification of Papile et al. will be used to classify the severity of IVH [16, 17]; diagnosis of PVL will be made in the presence of cystic areas detected with brain ultrasound at 40 post-conception weeks [18, 19]; ROP will be evaluated in accordance with the International ROP Classification [20]; diagnosis of NEC will be made according to Bell criteria [21]. The diagnosis of sepsis will be based on clinical and laboratory data (total neutrophil count, immature/total neutrophil ratio, concentration of C-reactive protein) confirmed by the presence of at least one positive blood or liquor culture.

Each newborn will be identified with an alphanumeric code (pseudo-anonymization procedure) to guarantee data confidentiality.

In the following, the study period time table is reported (Table 1).

**Table 1:** Study period timetable

**Timepoint**	**Study period**						
	**Enrolment**	**Allocation**	**Post-allocation**				**Close-out**
	***Prenatal or postnatal before 180 min of life***	**Postnatal before 180 min of life**	***0–180 min of life***	***24*** ± **2 h of life**	***1 week* ± 1 day of life**	***36 weeks of GA or earlier if patient is discharged***	***Discharge***
**Enrolment**							
**Eligibility screen**	X						
**Informed consent**	X						
**Allocation**		X					
**Interventions**							
***LUS group*** ***Lung ultrasound and FiO2 evaluation for surfactant need***			X				
***Control group*** ***FiO2 evaluation for surfactant need***			X				
**Assessments**							
***Baseline data*** *** Delivery data***			X				
***Clinical data***			X	X	X	X	
***Lung ultrasound score***			X	X	X	X	
***Blood Gas Analysis (if available, performed ONLY for normal clinical practice)***			X	X	X	X	
***Clinical Outcomes***							X

### Data collection

All study data will be obtained from clinical records and will be electronically entered by the local principal and sub-investigators at each participating site where the data originated, using a validated web-based system. Data integrity will be enforced through appropriate range checks and consistency checks at the time of data entry, before the data are committed to the database. Additional errors will be detected by programs designed to detect missing data or specific errors in the stored data. These errors will be summarized along with detailed descriptions for each specific problem in a data query report, which will be sent to the principal investigator of each site. Data entered into the database will be retrievable for viewing through the data entry application. The audit trail will register all the activities performed by the authorized operators. The type of activity that an individual user may undertake is regulated by the privileges associated with his/her user identification code and password. A complete back up of the primary database will be performed once a week and stored indefinitely on a twin server. Incremental data back-ups will be performed on a daily basis and retained for at least 1 week on-site.

There was no structured plan to promote participant retention.

### Statistical analysis

Clinical characteristics of infants in the CG and LUSG will be described using mean values and standard deviation, median and interquartile range, or rate and percentage. Univariate statistical analysis will be performed using the Student’s *t* test for parametric continuous variables, the Wilcoxon rank-sum test for non-parametric continuous variables, and Fisher’s exact test for categorical variables. A *p* value < 0.05 will be considered statistically significant.

Subsequently, clinical characteristics which are most likely associated with BPD or death will be included in a multiple logistic regression analysis to assess their independent role in predicting clinical outcome. Effect estimates will be expressed as odds ratios (ORs) with maximum likelihood-based 95% confidence limits.

The primary statistical analyses will be performed according to the intention-to-treat principle. The per-protocol analyses will be also performed with an explanatory intent. Major protocol deviations will be reported. Patients without any information available about the primary study endpoint will be classified as failure. Single imputation will be used in case of missing values will be observed for the covariates included in the adjusted and subgroup analyses.

### Additional analyses

An interim analysis will be planned after 167 infants are enrolled for each arm (334 total infants enrolled). The analysis will aim to compare treatment arms with respect to efficacy, safety, futility, and, if necessary, a sample size adjustment. In the interim, the prespecified stopping rules for safety are: a mortality rate > 40%, a rate of severe IVH > 30%, and a pneumothorax rate of > 10% in the LUSG compared to CG. The interim analysis will be performed by an independent statistician, blinded for treatment allocation. The statistician will report to the independent Data and Safety Monitoring Board (DSMB) which will have unblinded access to all data and will discuss the results of the interim analysis with the Steering Committee in a joint meeting. The Steering Committee will decide whether to continue the trial and will report to the central Ethics Committee.

### Definition of study conclusion

The study will be considered concluded for each patient at the time of discharge or death or in case of withdrawal of consent. The study will be considered definitively concluded upon discharge or death or withdrawal of consent of the last patient enrolled.

### Withdrawal of subjects

Parent(s) or legal guardian(s) may withdraw consent to participate in the study at any time.

### Ethical considerations

This study will be conducted in accordance with the Declaration of Helsinki, Edinburgh revision (2000), with the directive CPMP/ICH/135/95, implemented by the Ministerial Decree of 15 July 1997 (Good Clinical Practice) and in accordance with the Ministerial Decree of 10 May 2001 published in the Official Gazette no. 139 of 18 June 2001. In this regard, the medical interventions and laboratory and instrumental analysis procedures envisaged by this protocol are normally considered to be of good clinical practice in patients of this type.

The protocol has been approved by the Tuscan Pediatric Ethical Committee protocol n. 302/2021, with amendment n. 278–2/2022.

### Quality control and quality assurance procedures

Compliance will be defined as full adherence to protocol. Compliance with the protocol will be ensured by several procedures as described below.

### Site set-up

Local principal investigators are required to participate in preparatory meetings in which details of study protocol, data collection, and procedures in control and lung ultrasound group will be accurately discussed. All centers will receive detailed written instructions on web-based data recording and, to resolve possible difficulties, the Clinical Trials Coordinating Center (Careggi University Hospital of Florence, Florence, Italy) will be available for assistance.

### Enrollment procedure

Obstetricians from the different hospitals will be aware of the study protocol and will inform the neonatologist of high-risk preterm births.

The study information leaflet will be provided and explained to parents, relatives, and guardians of eligible patients in the hours preceding birth by trained staff. Parents’ consent must be obtained within 180 min following birth and before the randomization procedure. In cases of spontaneous preterm labor and subsequent vaginal or cesarean delivery, the informed consent will be obtained immediately after birth.

Risks and benefits will be fully discussed. Parents will be informed that the study does not include blood sample or other interventions other than those routinely performed in the NICU.

Informed consent will be obtained by the principal investigator of each center or the collaborators in charge. The patients will be enrolled by the principal investigator or the collaborators in charge at the time of the delivery.

### Data processing and monitoring

All study data will be:

1. Screened for out-of-range data, with cross-checks made for conflicting data within and between data collection forms by a data manager.
2. Referred back to the relevant center for clarification in the event of missing items or uncertainty. A record of all discrepancies and resolutions will be kept by the data manager.

The chief investigator and trial statistician will review the results generated for logic and for patterns or problems. Outlier data will be investigated.

### Safety

Safety end-point measures will include incidence, severity, and causality of reported serious adverse events (SAEs), namely changes in the occurrence of expected common prematurity complications and clinical laboratory test assessments, and the development of unexpected SAEs in this high-risk population. All SAEs will be followed until satisfactory resolution is achieved or until the investigator responsible for the care of the participant deems the event to be chronic or the patient to be stable. All expected and unexpected SAEs, whether or not they are attributable to the study intervention, will be reviewed by the local principal investigators to determine if there is a reasonable suspected causal relationship to the intervention. If the relationship is reasonable, SAEs will be reported to chief investigators who will report to the Ethics Committee and request all investigators to guarantee the safety of all participants. There is no specific plan for auditing trial conduct.

### Dissemination policy

The data will be owned by the promoter and will be shared with the investigators. Ownership of study data will remain with the investigators involved. The results of the study will be published and may also be the subject of communications, reports, or posters at congresses. The results of the study will be made available for publication.

The promoter will be responsible for study design, supervision of the study and its conduct according to Good Clinical Practice, the final processing of the data, and dissemination of the study results.

The main investigators of each center will be responsible for recruiting patients and compiling the eCRF.

The principal investigator of the coordinating center will inform the ethics committee of the center of any modification to the study protocol in order to obtain a new approval. Subsequently, he will notify the principal investigator of each center and the respective ethics committees of this changes in order to obtain the local approval. There is currently no public access to the original protocol, dataset, or statistical code. The available information is currently deposited on the clinicaltrials.gov website. NCT05198375.

All presentations and publications are expected to protect the integrity of the major objectives of the study; data that break the blind will not be presented prior to the release of mainline results.

## Discussion

Functional LU has proven to be an important imaging technique in NICU to reduce radiation exposure [22, 23] and to provide longitudinal assessment of respiratory diseases in premature neonates [24, 25]. Moreover, ultrasound quantification of RDS severity and calculation of LUS has been proven to predict non-invasive respiratory support failure in neonates affected by RDS more accurately than FiO_2_ requirements or other clinical parameters. Thus, LUS has the potential to be incorporated into clinical decision-making at cot-side [3, 4] as an additional tool when deciding about surfactant replacement treatment, although there is still no consensus on which score and cut-off value is the most accurate [11].

Current European recommendations consider LU a useful adjunct to determine RDS severity in experienced hands [1]. However, there is not enough evidence available to support a more practical recommendation on LU, with indications regarding LU timing and LUS cut-off. Currently, the FiO_2_ criterion is the only quantitative criterion which the European guidelines rely on, in neonates with RDS on non-invasive respiratory support, although this raises several concerns. First, oxygenation is influenced not only by oxygen requirements but also by the mean airway pressure. Thus, regardless of RDS severity, the type and degree of respiratory support may significantly influence oxygen requirements. Second, other variables such as peripheral perfusion, temperature, and degree of right to left shunt due to delayed decrease of pulmonary arterial pressure can influence oxygen requirements. Third, given the fact that RDS is a progressive disease, neonates may fulfill the FiO2 criterion only in an advanced phase of the disease, outside the ideal therapeutic window for early rescue treatment (within the first 3 h of life). This is a major concern. In fact, there is a large body of evidence that this period is the ideal therapeutic window for SRT compared to later administration, which translates into clinical benefits, in particular decreased incidence of air leaks, mechanical ventilation, BPD, or death [2]. The incorporation of LUS into clinical decision-making has already proven to significantly increase the proportion of neonates receiving SRT within 3 h of birth [3, 4] although, as previously mentioned, no data are available from multicenter RCTs.

In conclusion, relying only on the FiO_2_ criterion to guide SRT in neonates with RDS on non-invasive respiratory support may have suboptimal accuracy.

Our hypothesis is that LUS may predict more accurately which preterm infants, assisted in nCPAP for RDS, may benefit from early surfactant administration. Instead of evaluating only the oxygen requirements, the use of semi-quantitative LU allows quantification of RDS severity in a score (LUS). LUS estimates the degree of lung aeration or lung recruitment, which reflects the amount of alveolar surface available for gas exchange. This hypothesis was confirmed by the ULTRASURF study [4]. The authors showed that patients randomized to SRT based on LUS received earlier treatment and with lower oxygen requirements compared to neonates who received SRT according to FiO_2_ value. However, the study has several limitations, such as the relatively small sample size, the moderate prematurity of studied infants, and the single center study design.

Therefore, we designed a study which overcomes these limitations. Specifically, we aimed to recruit a large cohort of patients and calculate a sample size powered to demonstrate an improvement in the composite clinical outcome of BPD or death; we stratified the recruitment for gestational age to include an adequate proportion of extremely preterm neonates; and we included several centers in different European countries to allow for an improved generalizability of the study results.

All the participating centers have considerable experience in LU, LUS calculation, and non-invasive respiratory support. Moreover, in the preliminary meetings, we discussed respiratory management during the first hours of life and reached a consensus in order to uniform and standardize the respiratory management among the participating centers as much as possible to limit possible sources of bias.

There are some limitations in our study design. First, the participating centers adopt different methods of surfactant delivery: LISA, INSURE, and IN-REC-SURE. Second, once the patient has received SRT, different respiratory strategies and devices are used to deliver non-invasive respiratory support among participating centers. However, although there is variability between centers, the same protocol is adopted within the center. Since we randomize on center, this is likely not to affect the differences between the two groups within the center.

## Trial status

Protocol version 3.0, date 25 September 2023. Recruitment began 5 April 2022. The trial is currently recruiting study subjects. Active center (25 September 2023): 18. Enrolled infants (25 September 2023): 213. Estimated end of recruitment: 31 December 2025.

## Data Availability

Not applicable.
